# The circadian clock regulates rhythmic activation of the NRF2/glutathione-mediated antioxidant defense pathway to modulate pulmonary fibrosis

**DOI:** 10.1101/gad.237081.113

**Published:** 2014-03-15

**Authors:** Vanja Pekovic-Vaughan, Julie Gibbs, Hikari Yoshitane, Nan Yang, Dharshika Pathiranage, Baoqiang Guo, Aya Sagami, Keiko Taguchi, David Bechtold, Andrew Loudon, Masayuki Yamamoto, Jefferson Chan, Gijsbertus T.J. van der Horst, Yoshitaka Fukada, Qing-Jun Meng

**Affiliations:** 1 Faculty of Life Sciences, University of Manchester, Manchester M13 9PT, United Kingdom;; 2 Department of Biophysics and Biochemistry, Graduate School of Science, University of Tokyo, Tokyo 113-0033, Japan;; 3 Department of Medical Biochemistry, Tohoku University Graduate School of Medicine, Sendai 980-8575, Japan;; 4 University of California at Irvine, Irvine, California 92697, USA;; 5 Department of Genetics, Center for Biomedical Genetics, Erasmus University Medical Center, 3000 CA Rotterdam, The Netherlands

**Keywords:** circadian clock, glutathione, NRF2, bleomycin, pulmonary fibrosis

## Abstract

The disruption of the NRF2 (nuclear factor erythroid-derived 2-like 2)/glutathione-mediated antioxidant defense pathway is a critical step in the pathogenesis of several chronic pulmonary diseases and cancer. While the mechanism of NRF2 activation upon oxidative stress has been widely investigated, little is known about the endogenous signals that regulate the NRF2 pathway in lung physiology and pathology. Here we show that an E-box-mediated circadian rhythm of NRF2 protein is essential in regulating the rhythmic expression of antioxidant genes involved in glutathione redox homeostasis in the mouse lung. Using an in vivo bleomycin-induced lung fibrosis model, we reveal a clock “gated” pulmonary response to oxidative injury, with a more severe fibrotic effect when bleomycin was applied at a circadian nadir in NRF2 levels. Timed administration of sulforaphane, an NRF2 activator, significantly blocked this phenotype. Moreover, in the lungs of the arrhythmic *Clock*^*Δ19*^ mice, the levels of NRF2 and the reduced glutathione are constitutively low, associated with increased protein oxidative damage and a spontaneous fibrotic-like pulmonary phenotype. Our findings reveal a pivotal role for the circadian control of the NRF2/glutathione pathway in combating oxidative/fibrotic lung damage, which might prompt new chronotherapeutic strategies for the treatment of human lung diseases, including idiopathic pulmonary fibrosis.

## Abstract (precis)

Disruption of the NRF2/glutathione-mediated antioxidant defense pathway is critical for the pathogenesis of chronic pulmonary diseases. Here, Pekovic-Vaughan et al. demonstrate that a circadian rhythm of NRF2 protein is essential in regulating the rhythmic expression of antioxidant genes in the mouse lung. When bleomycin was applied at a circadian nadir in NRF2 levels, a more severe lung fibrotic effect was observed. These findings reveal a crucial role for the circadian control of the NRF2 pathway in combating fibrotic lung damage.

In mammals and humans, the circadian clocks in the brain and periphery are responsible for generating ∼24-h daily rhythms that control physiology, metabolism, and behavior (Reppert and Weaver 2002; Hastings et al. 2003). At the molecular level, circadian rhythms emerge from the interlocked transcriptional/translational feedback loops consisting of core clock genes and oscillatory metabolic products (Ko and Takahashi 2006). The core clock transcription factors CLOCK and BMAL1 activate the expression of repressors *Period* (*Per*) and *Cryptochrome* (*Cry*) genes as well as a spectrum of clock-controlled genes (CCGs). Subsequently, accumulation of PER/CRY protein complexes inhibits the BMAL1/CLOCK activity, leading to rhythmic repression of their own transcription and of other CCGs. Many of the known CCGs encode rate-limiting enzymes and transcription factors that play pivotal roles in cellular metabolism and function. Nonetheless, the battery of genes under circadian regulation is largely tissue-specific so that circadian clocks modulate physiological processes unique to each organ (Panda et al. 2002; Gossan et al. 2013).

Daily changes in the environment (cycles in light/darkness, feeding, rest–activity, and temperature fluctuations) inevitably expose the mammalian cells and tissues to periodic challenges, including oxidative insults from environmental toxins/pollutants and endogenously produced reactive metabolites as products of respiration (Patel et al. 2014). Failure to scavenge reactive oxygen species (ROS), reactive nitrogen species (RNS), and reactive electrophiles results in excessive oxidative stress, leading to damage to critical macromolecules and cellular structures (Veal et al. 2007). The ability to anticipate and withstand such cyclic insults (e.g., through effective ROS scavenging) is therefore essential for normal protective tissue functions. There is emerging evidence that circadian clocks regulate processes that keep ROS at physiological levels and protect organisms from oxidative stress (Stangherlin and Reddy 2013; Patel et al. 2014). Indeed, self-sustained rhythms in the cellular redox state (Kondratov et al. 2006; O'Neil et al. 2011; Edgar et al. 2012; Wang et al. 2012) as well as ROS-responsive antioxidant genes (Geyfman et al. 2012; Lai et al. 2012) have been observed in the brain and/or peripheral tissues. Moreover, diurnal rhythms in organismal survival to ROS-generating agents have been reported (Krishnan et al. 2008). However, the underlying molecular mechanisms of clock-controlled regulation of protective antioxidant responses and their role in tissue-specific physiology and pathology are still not well understood.

One of the major cellular antioxidant defense pathways is mediated through the cap'n'collar basic leucine zipper transcription factor NRF2 (nuclear factor erythroid-derived 2-like 2). The current paradigm of NRF2 regulation has mainly focused on oxidative stress-induced post-translational mechanisms, including regulation by its negative regulators (KEAP1, BACH1, and β-TrCP), positive regulators (DJ1, p62, and p21), or protein modifications (Itoh et al. 1999; McMahon et al. 2003; Chen et al. 2009; Komatsu et al. 2010; Chowdhry et al. 2013). In response to oxidative stress, NRF2 protein translocates to the nucleus and binds to the antioxidant response elements (AREs) in the promoters of many antioxidant genes. Subsequently, NRF2 induces a transcriptional program that maintains cellular redox balance and protects cells from oxidative insults (Reddy et al. 2007; Malhotra et al. 2010). NRF2 transcriptional targets include glutathione cysteine ligase (GCL), involved in the rate-limiting step of biosynthesis of antioxidant glutathione; glutathione-utilizing enzymes such as glutathione S-transferase (GST); and haeme oxigenase 1 (HMOX1), involved in the catabolism of pro-oxidant haeme.

*Nrf2*-deficient mice show a decrease in both constitutive and inducible expression of multiple glutathione-dependent enzymes (McMahon et al. 2001), associated with an impaired antioxidant defense and susceptibility to stress-induced tissue pathologies (Cho et al. 2006; Chan and Kan 1999). Consequently, they are highly sensitive to many environmental toxicants and chemical inducers of oxidant injury and fibrosis in several tissues, including bleomycin-induced and butylated hydroxytoluene (BHT)-induced pulmonary fibrosis (Chan and Kan 1999; Cho et al. 2004; Kikuchi et al. 2010). In humans, altered NRF2 expression and impaired redox balance have been associated with the pathogenesis of chronic lung diseases (asthma, COPD, and idiopathic pulmonary fibrosis) as well as lung cancer (Cho et al. 2006; Hayes and McMahon 2009). Indeed, pharmacological targeting of NRF2 has emerged as a novel therapeutic approach to combat oxidative stress seen in chronic lung diseases (Malhotra et al. 2011; Artaud-Macari et al. 2013).

Recently, mRNA levels of *Nrf2* as well as several of its target genes have been reported to show diurnal variation in mouse livers and *Drosophila* heads (Beaver et al. 2012; Xu et al. 2012). However, whether and how endogenous circadian signals control NRF2-dependent antioxidant activity in tissue physiology and upon pathological challenges, especially in the lung, has not been defined. Here we investigated the circadian rhythms in the protein levels of NRF2 and activity of the NRF2/GSH pathway in lung tissues from light/dark cycle-entrained mice. We also report the physiological and pathological importance for the circadian control of the NRF2/GSH pathway using a well-established lung fibrosis model following a bleomycin challenge. Our data reveal a pivotal role for endogenous NRF2 rhythms in the lung in coupling protective antioxidant responses to the time-of-day susceptibility to oxidant injury and pulmonary fibrosis.

## Results

### Circadian protein expression of NRF2 transcription factor in the mouse lung

**Figure 1.**
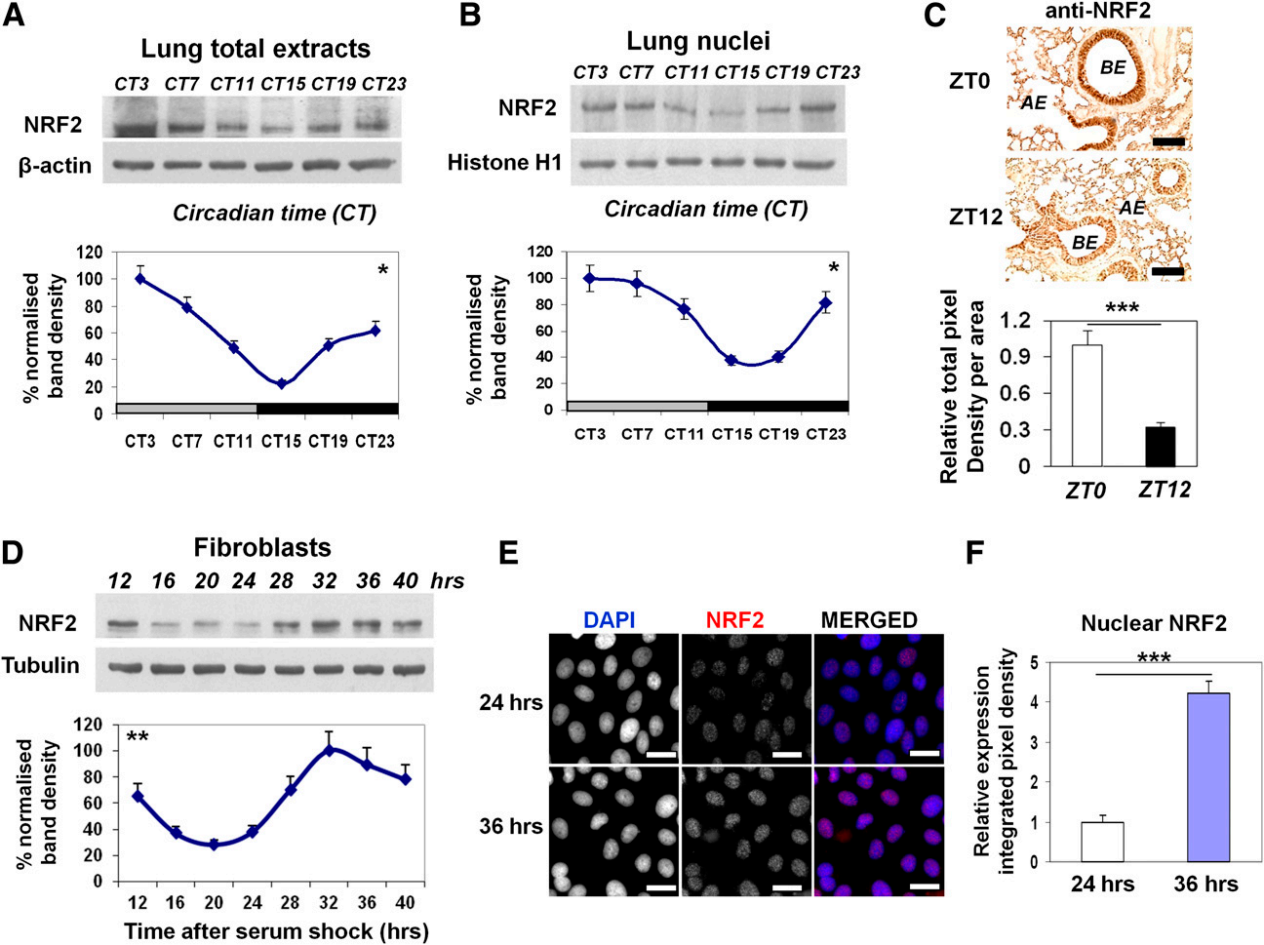
Circadian expression of NRF2 protein in mouse lungs and fibroblast cells. (*A*,*B*) Representative Western blots of rhythmic NRF2 levels in total lung lysates and nuclear extracts from wild-type (WT) mice kept in DD. NRF2 densitometry (mean ± SEM) was normalized to β-actin or histone H1, and the highest expression set as 100%. (Gray bar) Subjective day; (black bar) subjective night. One-way ANOVA for the effect of time, (*) *P* < 0.05. (*C*) Representative micrographs of histological lung sections showing temporal NRF2 immunoreactivity at ZT0 versus ZT12. Brown NRF2 staining was localized mainly to bronchial epithelium (BE) and alveolar epithelium (AE). Mean ± SEM; (***) *P* < 0.001, *t*-test. Bar, 100 μM. (*D*) Representative Western blots of temporal NRF2 levels in total lysates of Rat1 fibroblasts following serum shock. NRF2 densitometry (mean ± SEM) was normalized to tubulin. Harvest of cells started at 12 h after serum shock to exclude the initial gene induction. (**) *P* < 0.01, one-way ANOVA for the effect of time. (*E*, *F*) Representative immunofluorescent micrographs and quantification of endogenous NRF2 in Rat1 cells at 24 h or 36 h after serum shock. Nuclei were counterstained with DAPI. Bar, 50 μM. (***) *P* < 0.001, *t*-test.

To investigate the hypothesis that the NRF2-mediated antioxidant pathway is under circadian regulation in the lung, we first examined the total NRF2 protein levels in mouse lungs harvested at 4-h intervals from mice kept in constant darkness (DD). Western blotting using a specific NRF2 antibody (confirmed in *Nrf2* knockout mouse embryonic fibroblasts [MEFs]) (data not shown) revealed a robust rhythmic pattern of NRF2 protein (one-way ANOVA, Tukey test, *P* < 0.05). The NRF2 protein peaked at the circadian time 3–7 (CT3–CT7), reaching a trough at CT15–CT19 (Fig. 1A). Nuclear accumulation of NRF2 protein is essential for its subsequent activation of downstream genes (Reddy et al. 2007; Nguyen et al. 2009). Indeed, we detected rhythmic levels of NRF2 protein in the nuclear lung fractions, with the maximal levels corresponding to the peak of the total pool of NRF2 protein (*P* < 0.05) (Fig. 1B). Immunostaining of NRF2 revealed a significant time-of-day difference in the lungs that was mainly localized to the bronchial and alveolar epithelium, with an approximately threefold weaker signal at “zeitgeber” time 12 (ZT12; lights off) compared with ZT0 (lights on) (Fig. 1C). The rhythmic NRF2 protein expression was also evident in the total cell lysates (Fig. 1D) and nuclei (Fig. 1E,F) of clock-synchronized Rat1 fibroblasts, demonstrating the cell-autonomous nature of the NRF2 protein rhythms.

### The circadian clock exerts transcriptional control of the Nrf2 gene via a putative E-box element in its promoter

**Figure 2.**
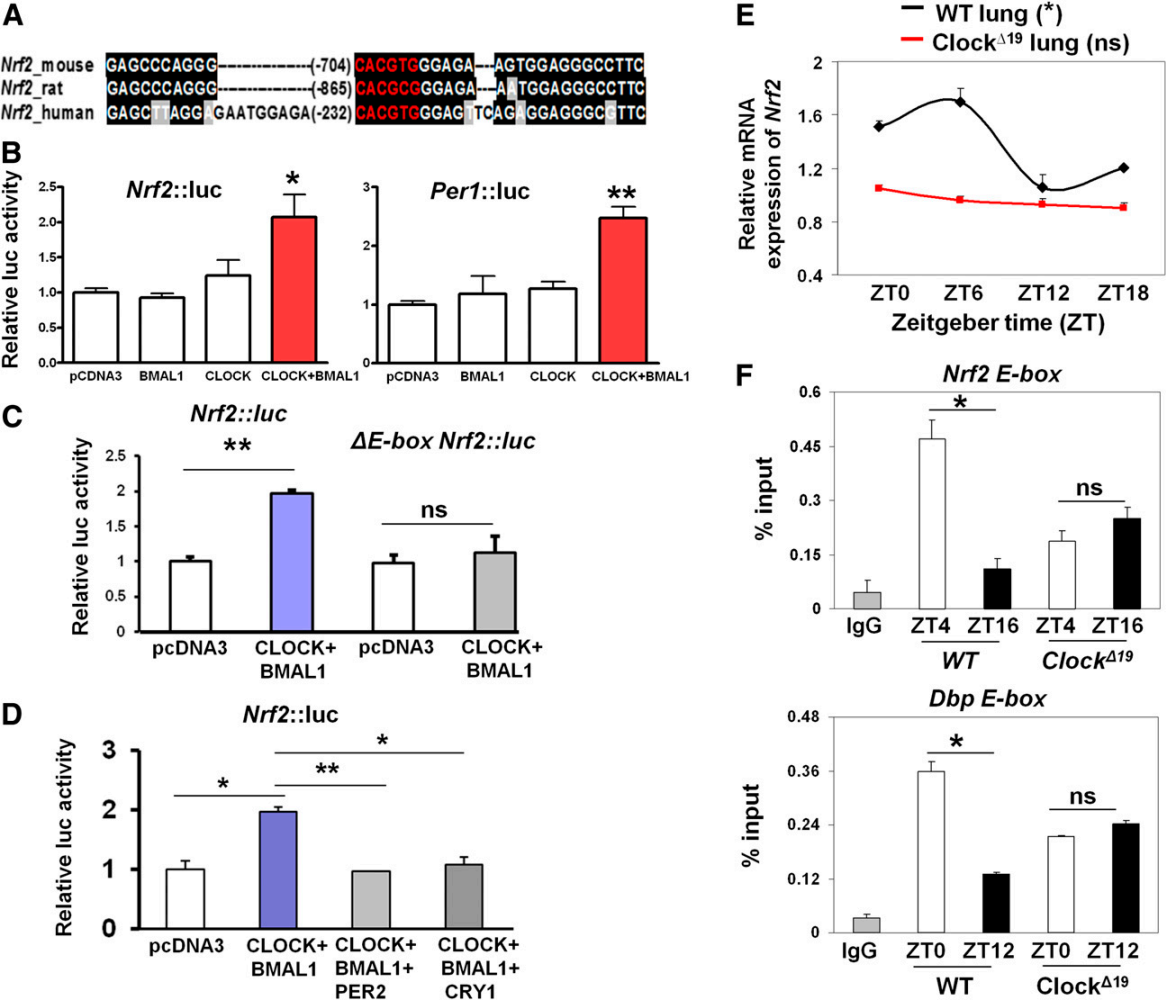
Circadian clock components control *Nrf2* transcription via a putative E-box element in vitro and in vivo. (*A*) A conserved putative E-box element (shown in red) in the proximal promoters of the mouse, rat, and human *Nrf2* gene. Sequences were aligned using the Clustal W algorithm and shaded with the Boxshade algorithm to highlight the conserved regions (shown in black). (*B*, *C*) Effects of CLOCK/BMAL1 overexpression on wild-type (WT) or ΔE-box *Nrf2*∷luc promoter activity in Rat1 cells. Data (mean ± SEM) were normalized to the β-galactosidase reporter gene and expressed relative to pCDNA3 control. The *Per1*∷luc reporter served as a positive control. (*) *P* < 0.05; (**) *P* < 0.01; (ns) not significant, *t*-test. (*D*) Effects of PER2 or CRY1 overexpression in suppressing the CLOCK/BMAL1-activated *Nrf2*∷luc promoter activity in Rat1 cells. (*) *P* < 0.05; (**) *P* < 0.01, *t*-test. (*E*) Temporal mRNA expression of *Nrf2* in wild-type and *Clock*^Δ19^ lungs. Data (mean ± SEM) were normalized to *Gapdh*. (*) *P* < 0.05 for wild-type lungs;( ns) not significant for *Clock*^Δ19^ lung; one-way ANOVA for the effect of time. (*F*) Temporal CLOCK binding to E-boxes in *Nrf2* and *Dbp* gene promoters in wild-type and *Clock*^Δ19^ lungs using CLOCK-specific ChIP. IgG served as a negative control. Data (mean ± SEM) were expressed as percent input. (*) *P* < 0.05; (ns) not significant, *t*-test. (White bar) Light phase; (black bar) dark phase.

The circadian clock transcription factors regulate target genes through the *cis*-acting elements in their gene promoters (Panda et al. 2002; Rey et al. 2011). We therefore analyzed the proximal promoter regions of the mouse, rat, and human *Nrf2* genes for clock-regulated transcriptional elements, which revealed a conserved putative E-box (CACGTG) close to their transcriptional start sites (TSSs; −704 base pairs [bp] relative to the TSS in the mouse *Nrf2*) (Fig. 2A). Next, we cloned the wild-type mouse *Nrf2*∷luc promoter (1.7 kb) and generated an E-box mutated construct. Coexpression of circadian transcription activators BMAL1 and CLOCK in Rat1 cells led to a significant stimulation of *Nrf2*∷luc activity (2.1-fold; *P* < 0.05), similar to the activation of the *Per1*∷luc promoter (containing three canonical E-box elements; 2.4-fold; *P* < 0.01) (Fig. 2B). Mutation of the core E-box sequence in the *Nrf2* promoter completely abolished its induction by CLOCK/BMAL1 complexes (Fig. 2C). Moreover, coexpression of circadian transcription repressors, either PER2 or CRY1, suppressed the CLOCK/BMAL1 transcriptional activation (Fig. 2D). Finally, stable transfection of the *Nrf2*∷luc reporter into Rat1 cells revealed a clearly discernible circadian rhythm with a period of 23.7 h (data not shown).

We next examined the temporal profiles of endogenous *Nrf2* mRNA in the lungs of wild-type and *Clock*^*Δ19*^ mice (a model in which the positive arm of the clock is genetically disrupted) (Vitaterna et al. 1994). This revealed a clear rhythmic expression of *Nrf2* transcripts in wild-type lungs (*P* < 0.05, one way ANOVA) (Fig. 2E). In contrast, the mRNA rhythm of *Nrf2* was abolished and remained low across all time points in the lungs of *Clock*^*Δ19*^ mice, consistent with a loss of rhythms in the clock gene expression (Fig. 2E; Supplemental Fig. S1). Consistently, knockdown of *Bmal1* by siRNA significantly reduced the levels of *Nrf2* mRNA in Rat1 fibroblasts (*P* < 0.05) (Supplemental Fig. S2). In contrast, in synchronized MEFs from arrhythmic *Cry1*^−/−^/*Cry2*^−/−^ mice, a model in which the negative arm of the clock is disrupted (Supplemental Fig. S3A,B; van der Horst et al. 1999), we observed constitutively high protein expression levels of NRF2 as well as mRNA levels of its two target genes (*Gclm* and *Gsta3*) (Supplemental Fig. S3C,D).

To verify the function of the putative E-box element in the *Nrf2* promoter in vivo, temporal chromatin immunoprecipitation (ChIP) assays were performed in the wild-type lungs using a CLOCK-specific antibody (Yoshitane et al. 2009). As a positive control, we first observed a time-dependent CLOCK binding to the E-box region of the *Dbp* promoter, a known CLOCK target (ZT10 > ZT22; *P* < 0.05) (Fig. 2F), as reported previously in the liver (Ripperger and Schibler 2006). Rhythmic CLOCK occupancy was also observed in the E-box region of the *Nrf2* promoter (Fig. 2F) but not in the distal region of the promoter (Supplemental Fig. S4A). There was a significantly higher binding of CLOCK at ZT4 as compared with ZT16, coinciding with the peak and trough expression of *Nrf2* mRNA, respectively. Moreover, CLOCK binding to the *Nrf2* promoter was arrhythmic and remained at an intermediate level in the *Clock*^*Δ19*^ lungs (Fig. 2F). Together, these results demonstrate that the *Nrf2* gene is directly regulated by the core clock components in vitro and in vivo through the conserved E-box element in its promoter.

### Rhythmic promoter binding of NRF2 drives oscillations of ARE-regulated antioxidant genes involved in glutathione homeostasis

**Figure 3.**
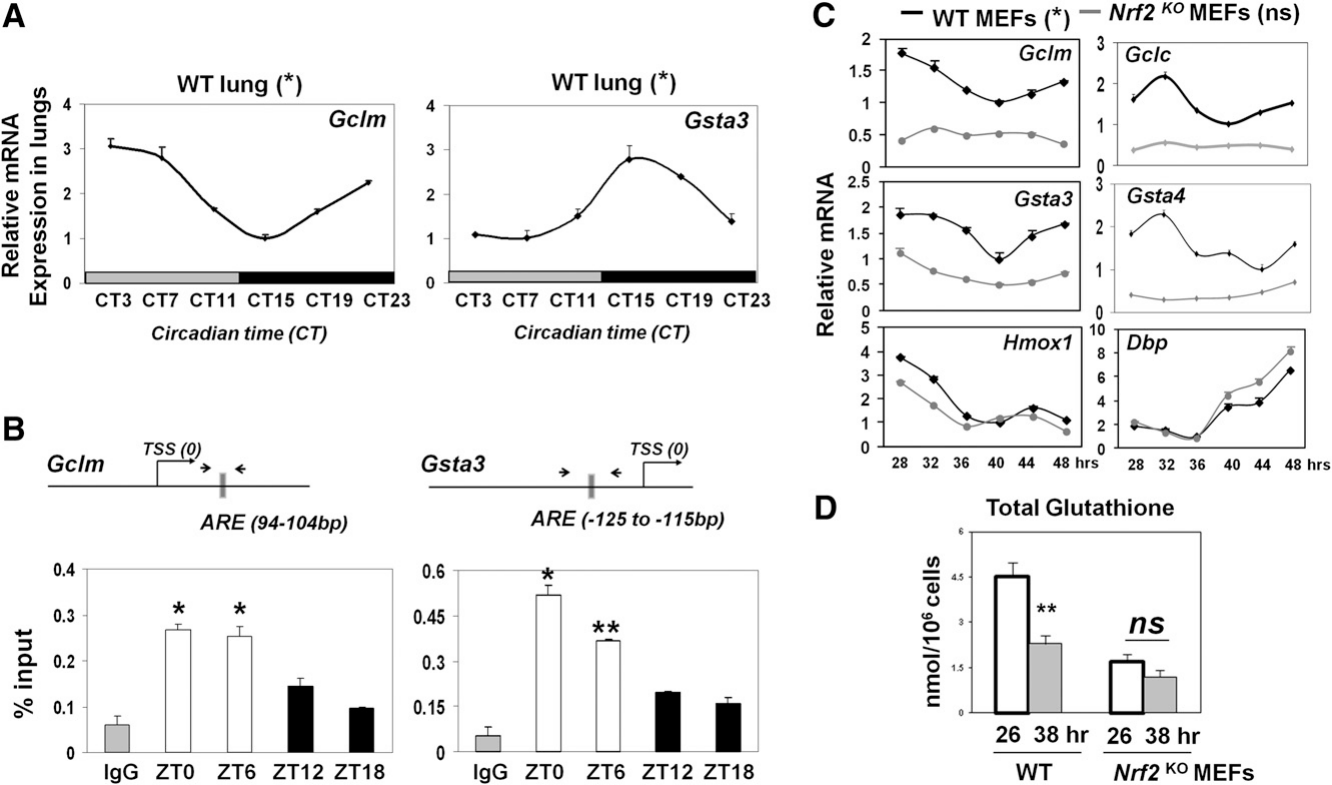
Circadian rhythm of NRF2 binding to ARE drives rhythmic oscillations of genes involved in glutathione synthesis and utilization. (*A*) Temporal mRNA levels of NRF2 targets in wild-type (WT) mouse lungs (DD). Data (mean ± SEM) were normalized to *Gapdh*, and the lowest expression was set as 1. (*) *P* < 0.05, one-way ANOVA. (*B*) Rhythmic NRF2 occupancy on AREs of antioxidant gene promoters in wild-type mouse lungs by NRF2-specific ChIP. The position of each ARE in relation to the TSS is shown. Mean ± SEM. (*) *P* < 0.05; (**) *P* < 0.01, *t*-test. (White bars) Light phase; (black bars) dark phase. (*C*) Temporal mRNA profiles of NRF2 target genes and clock gene *Dbp* in MEFs from wild-type and *Nrf2* knockout (KO) mice following serum shock. Harvest of cells started at 28 h after serum shock. Data (mean ± SEM) were normalized to *Gapdh*. (*) *P* < 0.05 for all six genes in wild type; not significant for *Nrf2* knockout MEFs (except *Hmox1* and *Dbp*; [*] *P* < 0.05), one-way ANOVA for the effect of time. (*D*) Temporal levels of reduced glutathione (GSH) in MEFs from wild-type and *Nrf2* knockout mice following serum shock. Data (mean ± SEM) were normalized to cellular counts and quantified using standard curve for reduced GSH. (**) *P* < 0.01; (ns) not significant, *t*-test.

Upon nuclear translocation, NRF2 protein binds to the AREs in the promoters of its target genes and thus maintains both basal and inducible expression of many antioxidant genes (Nguyen et al. 2009; Malhotra et al. 2010). The rhythmic nuclear NRF2 levels prompted us to examine whether the expression of ARE-regulated NRF2 target genes is also rhythmic. Temporal quantitative PCR (qPCR) of the mouse lungs demonstrated robust endogenous circadian rhythms for a number of NRF2 target genes, including *Gclm*, *Gsta3*, and *Hmox1* (*P* < 0.05) (Fig. 3A; Supplemental Fig. S5), as well as a core clock gene, *Bmal1* (*P* < 0.001) (Supplemental Fig.S5). To investigate the hypothesis that NRF2 may be rhythmically recruited to the AREs in the promoters of antioxidant genes in vivo, we performed temporal ChIP assays in the mouse lungs using an anti-NRF2 antibody. Here, we detected strongly rhythmic NRF2 binding specifically to the promoter regions spanning the well-characterized AREs (Malhotra et al. 2010) in *Gclm* and *Gsta3* gene promoters (Fig. 3B; Supplemental Fig. S4B). We observed significantly higher promoter occupancy at ZT0 and ZT6 (light phase) and lower occupancy at ZT12 and ZT18 (dark phase). The peak of NRF2 binding during light phase is in agreement with its maximal nuclear expression and the peaks of *Gclm* and *Hmox1* mRNAs in the lung. Interestingly, despite maximal NRF2 binding to the *Gsta3* promoter during the light phase, *Gsta3* mRNA peaked during early subjective night, suggesting that additional phase delay mechanisms may be at play, such as the availability of cofactors, as shown for the transcription factor BMAL1 (Rey et al. 2011), or post-transcriptional processing (Patel et al. 2014). The difference in the timing of maximal expression may be necessary for the specific function of individual NRF2 targets.

To investigate whether NRF2 is required for the rhythmic expression of antioxidant genes, we performed temporal qPCR for a panel of known NRF2 target genes in clock-synchronized MEFs from wild-type and *Nrf2* knockout mice. Here, we observed significantly reduced basal expression of many antioxidant genes in *Nrf2* knockout MEFs, as reported previously (Malhotra et al. 2010). Interestingly, the rhythmic patterns of genes coding for enzymes involved in glutathione synthesis (*Gclm* and *Gclc*) and glutathione utilization (*Gsta3* and *Gsta4)* were either severely dampened or abolished (Fig. 3C). In contrast, *Hmox1,* an inducible (but not basal) NRF2 target, remained robustly rhythmic in *Nrf2* knockout MEFs. Consistent with these results, the level of total glutathione (GSH) showed a significant temporal variation in wild-type MEFs but remained constitutively low in *Nrf2* knockout MEFs (Fig. 3D). Importantly, the rhythmic expression of *Dbp* in *Nrf2* knockout MEFs (Fig. 3C) and the behavioral free-running rhythms appear preserved in *Nrf2* knockout mice (Supplemental Fig. S6). These findings support a direct NRF2-dependent rhythmic control of its downstream targets rather than due to feedback actions of NRF2 on the molecular circadian clock. All together, these data demonstrate that the periodic NRF2 activity regulates the rhythmic GSH homeostasis through temporally controlling a key set of genes involved in glutathione synthesis and utilization.

### Time-of-day-dependent lung response to bleomycin challenge is coupled to temporal NRF2 activity

**Figure 4.**
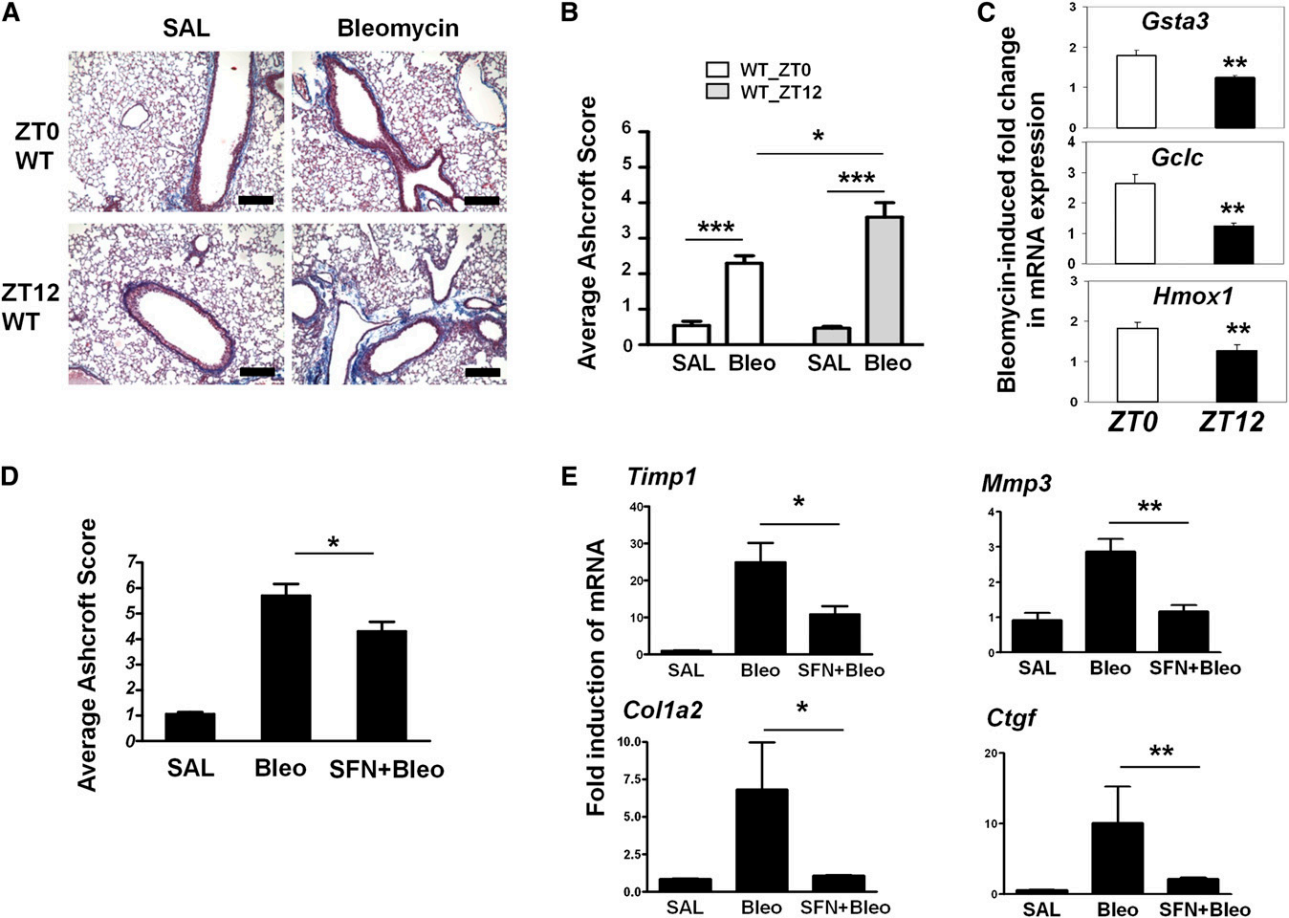
Time-of-day-dependent lung response to bleomycin challenge is coupled to temporal NRF2 activity. (*A*) Representative mouse lung sections were stained with Masson's Trichrome solution to reveal collagen fiber deposition (blue staining) and overall fibrosis. Wild-type (WT) mice were treated with bleomycin (*n* = 8) or saline (*n* = 5) at either ZT0 or ZT12 and, 7 d later, were sacrificed for tissue processing. Bar, 200 μM. (*B*) Lung sections in *A* were scored for fibrosis according to the Ashcroft scoring system. Mean ± SEM; (***) *P* < 0.001, saline versus bleomycin, *t*-test; (*) *P* < 0.05, ZT0 versus ZT12, two-way ANOVA. (*C*) Fold induction in wild-type lung mRNA levels of NRF2 targets (*Gclc*, *Gsta3*, and *Hmox1*) following bleomycin delivered at ZT0 or ZT12. Data (mean ± SEM) were expressed relative to saline controls at the respective time points. (**) *P* < 0.01, two-way ANOVA. (*D*,*E*) SFN treatment partially rescues the lung fibrosis phenotype, as revealed by Masson's Trichrome staining (with lower Ashcroft score) and reduced gene induction for fibrotic markers (*Timp1*, *Col1a2*, *Mmp3*, and *Ctgf*). Wild-type mice were treated with either saline (*n* = 3), bleomycin (*n* = 4), or SFN plus bleomycin (*n* = 5) and, 7 d later, were sacrificed for histological and gene expression analysis. Mean ± SEM; (*) *P* < 0.05; (**) *P* < 0.01, *t*-test.

The NRF2 antioxidant pathway is known to play a critical role in combating fibrotic injury (Cho et al. 2004; Walters and Kleeberger 2008). We therefore hypothesized that the rhythmic NRF2 activity may gate the tissue-protective responses to a fibrotic challenge in a time-of-day-dependent manner. To test this, we used an established in vivo mouse model of bleomycin-induced lung fibrosis (Walters and Kleeberger 2008). Bleomycin was administered intratracheally close to either the peak or nadir (ZT0 or ZT12, respectively) of NRF2 protein expression (see Fig. 1A–C). As expected, 7 d after bleomycin challenge, characteristic histological changes, including areas of inflammatory cell infiltration and alveolar wall thickening (data not shown), were observed at both time points. In addition, we also observed a significant increase in collagen accumulation around the peri-bronchial regions, as revealed by Masson's Trichrome staining of lung sections and quantification of fibrosis using the Ashcroft scoring system (Fig. 4A; Ashcroft et al. 1988). However, the degree of lung fibrosis was clearly dependent on the time of treatment and inversely correlated with NRF2 protein levels. A significantly higher fibrotic score was observed when bleomycin was delivered at ZT12 as compared with ZT0 (Fig. 4B), associated with higher induction of fibrotic gene markers (*Timp1*, *Col1a2*, and *Mmp3*) (Supplemental Fig. S7). Bleomycin challenge is known to activate NRF2-dependent antioxidant tissue responses (Cho et al. 2004). Following an acute bleomycin challenge (4 h), the induction of mRNAs for *Gclc*, *Gsta3*, and *Hmox1* showed a clear time-of-day dependence, with a significantly higher induction at ZT0 than at ZT12 (*P* < 0.01 for all three genes) (Fig. 4C), consistent with the hypothesis of a clock “gated” antioxidant tissue response.

To further establish the role of NRF2 rhythms in time-of-day susceptibility to bleomycin-induced fibrosis, we investigated whether the timed administration of sulforaphane (SFN; a direct NRF2 activator) can reduce the degree of bleomycin-induced fibrosis seen at the circadian nadir in NRF2 levels. To this end, wild-type mice were pretreated with either vehicle or SFN administered intraperitoneally at ZT6 (and thereafter at the same time point on days 1, 3, and 5) prior to a single challenge with bleomycin at ZT12 and assessed 7 d later. As a control, mice treated with SFN alone (at ZT6) showed increased NRF2 protein levels in the lungs at ZT12 as compared with vehicle treatment (data not shown). Here, the degree of bleomycin-induced lung fibrosis was significantly reduced by the timed SFN treatments (*P* < 0.05) (Fig. 4D). Consistent with these findings, the bleomycin-induced expression of fibrotic markers (*Col1a2*, *Timp1*, *Mmp3*, and *Ctgf*) was significantly blunted by SFN treatment (Fig. 4E). All together, these results strongly support a role for NRF2 rhythms in coupling antioxidant response to time-of-day susceptibility to bleomycin-induced lung fibrosis. Moreover, these findings suggest that timed SFN treatment might protect the lungs from oxidative challenges, especially those that fall within the nadir of NRF2 activity.

### Altered NRF2/GSH pathway activity in the Clock^Δ19^ lungs is associated with increased markers of oxidative damage and fibrotic injury

**Figure 5.**
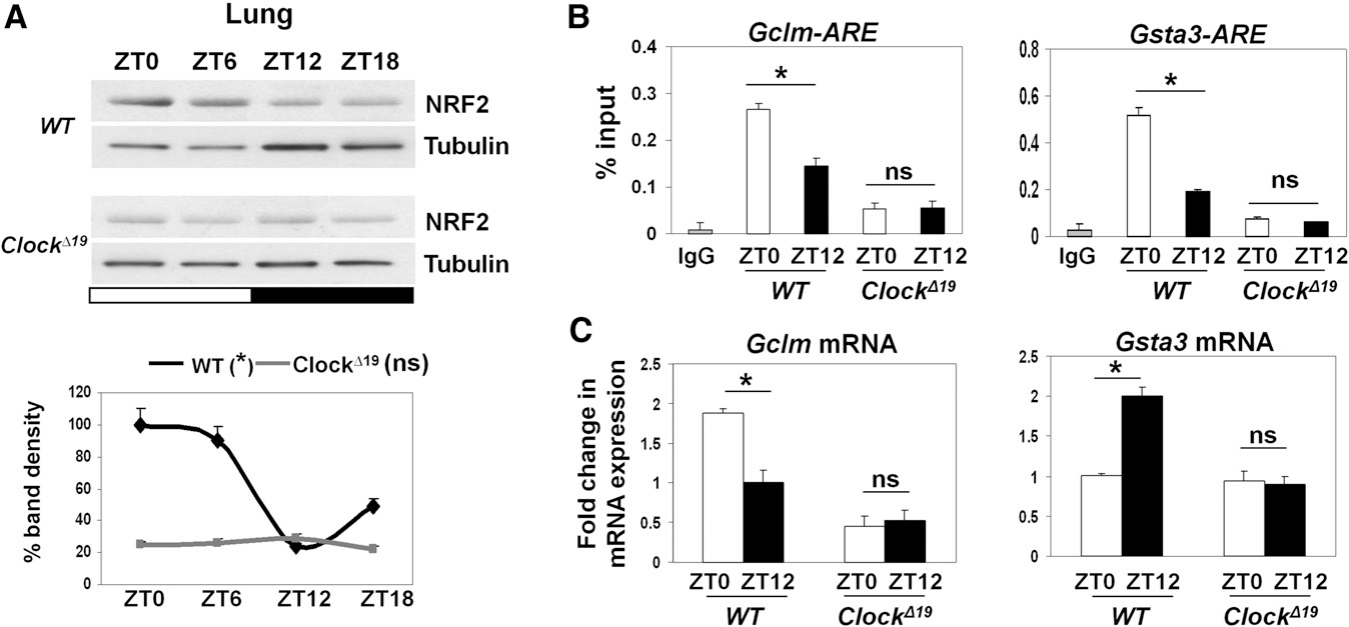
Loss of rhythmic NRF2 pathway activity in *Clock*^Δ19^ lungs. (*A*) Representative Western blots of diurnal NRF2 levels in total lung lysates from wild-type (WT) and *Clock*^Δ19^ mice. NRF2 densitometry (mean ± SEM) was normalized to tubulin and expressed relative to wild-type ZT0. (*) *P* < 0.05 for wild type; not significant for *Clock*^Δ19^, one-way ANOVA for the effect of time. (White bar) Light phase; (black bar) dark phase. (*B*) Temporal (ZT0 vs. ZT12) ChIP assays for the ARE regions of NRF2 targets (*Gclm* and *Gsta3*) in wild-type and *Clock*^Δ19^ lungs using NRF2-specific antibody. Data (mean ± SEM) were expressed as percent input. (*) *P* < 0.05; (ns) not significant, *t*-test. (*C*) Temporal mRNA levels of *Gclm* and *Gsta3* in wild-type and *Clock*^Δ19^ lungs at ZT0 versus ZT12. Data (mean ± SEM) were normalized to *Gapdh*. (*) *P* < 0.05; (ns) not significant, *t*-test.

Our data indicate that rhythmic NRF2 activity is coupled to the clock gated protective lung responses. To further establish the requirement of the functional circadian clock, we used the *Clock*^*Δ19*^ mouse model to investigate changes in the NRF2 pathway activity. Consistent with the loss of *Nrf2* mRNA rhythmicity in the *Clock*^*Δ19*^ lungs, NRF2 protein expression was constitutively low and arrhythmic (Fig. 5A). Moreover, ChIP assays revealed an arrhythmic and low NRF2 occupancy in the ARE region of the promoters for *Gclm* and *Gsta3* (Fig. 5B). While qPCR revealed robust time-of-day-dependent mRNA expression of *Gclm* and *Gsta3* in wild-type lungs, their expression in the *Clock*^*Δ19*^ lungs remained constitutively lower, at levels similar to or below the trough expression in wild-type lungs (Fig. 5C).

**Figure 6.**
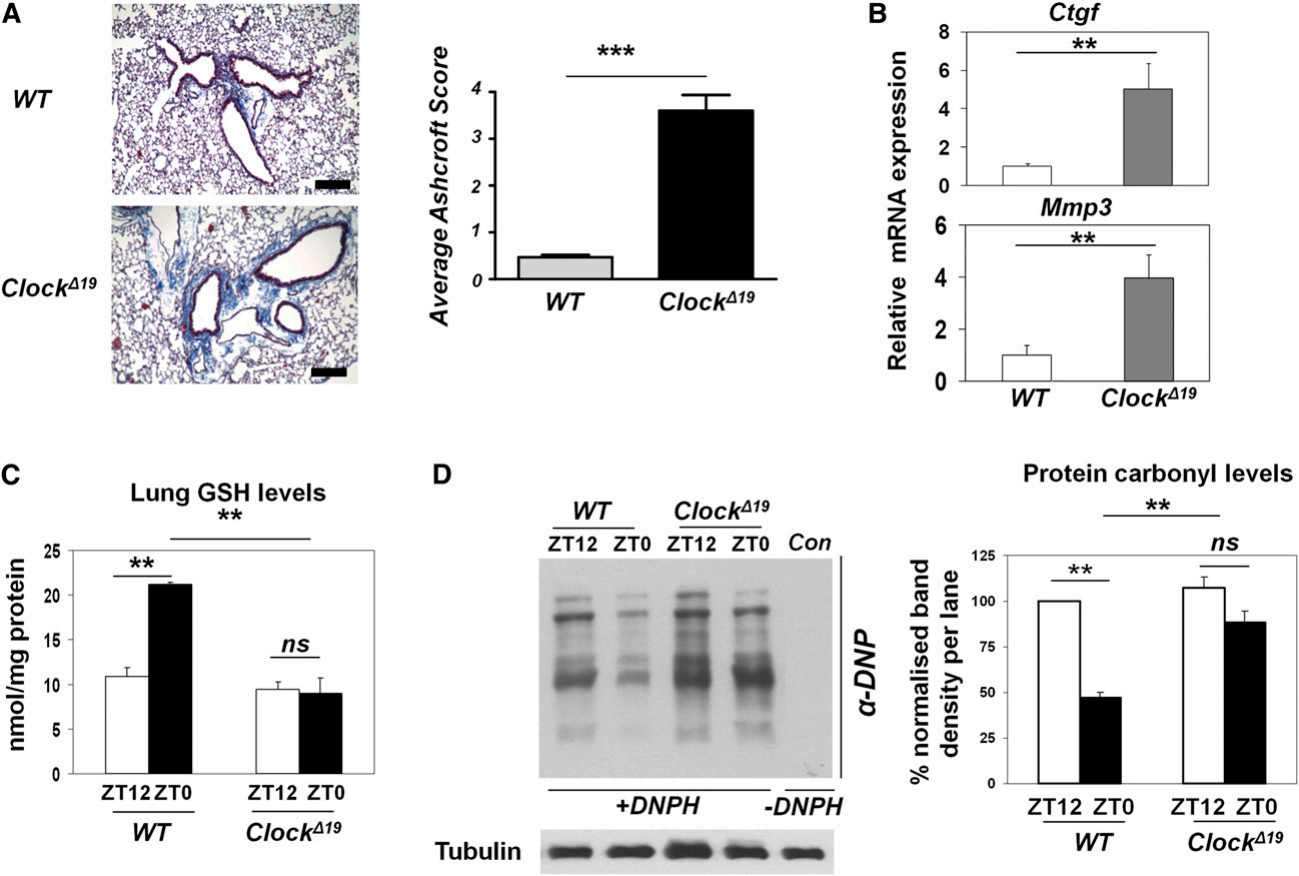
A fibrotic-like injury in the lungs of *Clock*^Δ19^ mice is associated with increased oxidative damage. (*A*) Representative wild-type (WT) and *Clock*^Δ19^ mouse lung sections processed for Masson's Trichrome staining. Bar, 200 μM. Quantification of lung fibrosis in wild-type (*n* = 4) and *Clock*^Δ19^ (*n* = 5) mice was performed by the Ashcroft scoring system. (***) *P* < 0.001, *t*-test. (*B*) mRNA levels of fibrotic markers (*Ctgf* and *Mmp3*) in wild-type versus *Clock*^Δ19^ lungs. Data (mean ± SEM) were normalized to *Gapdh*. (**) *P* < 0.01, *t*-test. (*C*) Temporal levels of reduced glutathione (GSH) in wild-type and *Clock*^Δ19^ lungs at ZT0 versus ZT12. Data (mean ± SEM) were normalized to total protein concentration and quantified using a standard curve for reduced GSH. (**) *P* < 0.01; (ns) not significant, *t*-test. (*D*) Protein carbonyl levels in wild-type and *Clock*^Δ19^ lungs at ZT0 versus ZT12 using oxy blotting with anti-dinitrophenyl (DNP) antibody. (*+*DNPH ) Proteins derivatized with 2,4-dinitrophenylhydrazine (DNPH); (−DNPH) proteins prepared in the absence of DNPH. Data (mean ± SEM) were normalized to tubulin and expressed relative to wild-type at ZT12. (**) *P* < 0.01; (ns) not significant, *t*-test.

In an attempt to test the hypothesis that the *Clock*^*Δ19*^ lungs were more prone to bleomycin-induced fibrotic injury, unexpectedly, we identified a spontaneous fibrotic-like phenotype in *Clock*^*Δ19*^ lungs even without a bleomycin challenge. We found a significantly increased deposition of collagen fibers around the bronchioles in *Clock*^*Δ19*^ compared with wild-type lungs (*P* < 0.001) (Fig. 6A). Consistently, the expression of fibrotic marker genes (*Mmp3* and *Ctgf*) was significantly elevated in *Clock*^*Δ19*^ lungs (Fig. 6B). A reduced GSH level is known to be involved in the fibrotic injury and remodeling processes (Liu and Gaston Pravia 2009). To explore the underlying mechanisms for the observed lung phenotype, we compared the temporal levels of reduced glutathione, a major antioxidant output of the NRF2 pathway. In wild-type lungs, we found a significantly higher level of reduced GSH at ZT0 compared with ZT12 (Fig. 6C). In contrast, *Clock*^*Δ19*^ lungs showed constitutively lower levels of reduced GSH, equivalent to the nadir level in wild-type lungs. Similar alterations in GSH level were also found in synchronized *Clock*^*Δ19*^ MEFs, which could partially be restored by SFN treatment (Supplemental Fig. S8; Higgins et al. 2009), supporting the involvement of NRF2. To determine whether the low GSH levels in *Clock*^*Δ19*^ lungs correlated with an increased oxidative burden, we temporally examined the lung protein carbonylation levels (a marker of oxidative damage). We observed a time-dependent change in the protein carbonyl levels in wild-type lungs, which were anti-phasic to the reduced GSH levels (Fig. 6D). In contrast, protein carbonylation levels remained high at both time points in *Clock*^*Δ19*^ lungs, consistent with a constitutive decline in GSH levels. These data support the hypothesis that the functional clock is required in coordinating the temporal activation of the NRF2/GSH pathway in the lung, disruption of which contributes to the increased susceptibility of the lung to cyclic oxidative stress and profibrotic challenges.

## Discussion

**Figure 7.**
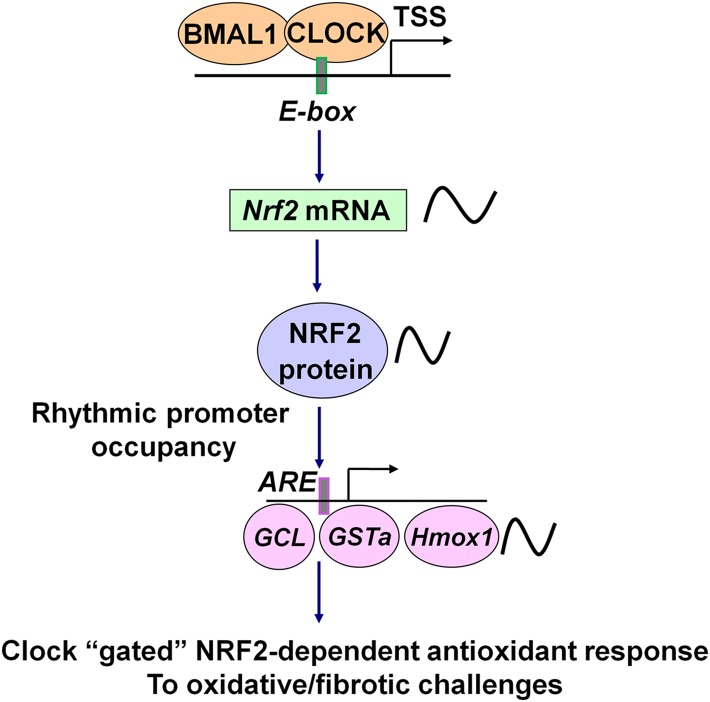
A hypothetical model depicting how the circadian clock exerts regulation of the NRF2 pathway under physiological conditions.

It has recently been suggested that circadian clocks may mediate the protective responses to oxidative stress (Stangherlin and Reddy 2013; Patel et al. 2014). However, the underlying clock-controlled molecular mechanisms and their role in tissue-specific physiology are still poorly understood. In this study, we identified the circadian clock as an endogenous molecular regulator of the NRF2/glutathione-mediated antioxidant defense pathway in the mouse lung, which couples the protective antioxidant response to the time-of-day variation in susceptibility to oxidant injury and pulmonary fibrosis. Based on our findings, we propose a working model depicting how the circadian clock exerts its regulation of protective antioxidant responses in the lung through the control of transcription factor NRF2 under physiological conditions (Fig. 7). The circadian transcription factors CLOCK and BMAL1 positively regulate *Nrf2* transcription through temporal control of the E-box element in the *Nrf2* gene promoter. NRF2 protein thus accumulates in a circadian manner and drives circadian transcription of a key set of antioxidant genes involved in glutathione metabolism (such as *Gclm* and *Gsta3*). The periodic NRF2 activity is exerted through its rhythmic recruitment to the AREs in the promoters of target antioxidant genes and mediates a time-coordinated protection against oxidative tissue injury and fibrotic damage.

Indeed, using an in vivo bleomycin-induced pulmonary fibrosis model, we show for the first time that, in the lung, the acute induction of the NRF2-mediated antioxidant response is temporally “gated” by the clock and coupled to a time-dependent resistance to fibrotic injury. Importantly, a scheduled pharmacological activation of NRF2 in vivo was able to significantly mitigate the decrease in resistance to fibrotic effects of bleomycin delivered at the circadian nadir in NRF2 levels. Moreover, in the lungs of *Clock*^*Δ19*^ circadian mutant mice, the altered temporal regulation of NRF2 antioxidant activity was associated with diminished glutathione levels, increased oxidative protein damage, and a spontaneous fibrotic-like phenotype. All together, our findings highlight an important role of the circadian clock in the regulation of the NRF2/GSH-mediated antioxidant response and its coupling to oxidative/fibrotic injury in the lung.

Our study builds on the recent reports implicating the role of the circadian clock in the regulation of tissue physiology through redox-dependent mechanisms (Kondratov et al. 2006; Lai et al. 2012; Wang et al. 2012; Musiek et al. 2013; Peek et al. 2013). For instance, direct circadian regulation of antioxidant genes (*Nqo1* and *Aldh2*) has been reported in the cerebral cortex, and the circadian regulation of NAD^+^-dependent histone deacetylase SIRT3 has been implicated in mitochondrial oxidative metabolism. Our findings reveal a novel aspect of circadian clock function in modulating pulmonary fibrotic responses, which is mediated through its control of the NRF2 transcription factor.

Current research concerning NRF2 regulation has largely focused on the post-translational mechanisms, especially via a KEAP1-based protein turnover in response to various electrophilic/oxidative stresses. In this model, NRF2 proteins are tethered to the cytoplasm by KEAP1 and targeted for proteasomal degradation (Hayes and McMahon 2009). It was therefore thought that only a small pool of NRF2 protein can escape from its degradation in the cytosol and translocate to the nucleus under basal conditions. In response to oxidative stress, however, oxidative inactivation of KEAP1 and de novo synthesis of NRF2 drive sustained nuclear NRF2 accumulation (Itoh et al. 2010). Our data reveal a rhythmic nuclear abundance of endogenous NRF2 protein in tissues and cells that accompanies the rhythmic levels in the total pool of NRF2 protein. These results are supported by previous studies in the mouse liver and pancreatic β cells that identified *Nrf2* as one of the clock-controlled targets (Xu et al. 2012; Lee et al. 2013). It is therefore conceivable that during the circadian peak of NRF2 protein, increased levels of NRF2 may saturate the binding capacity of KEAP1, thus allowing NRF2 to escape from the KEAP1-mediated degradation and translocate to the nucleus, where it induces higher expression of antioxidant genes. In contrast, during the nadir phase of NRF2 expression, KEAP1 may exert tighter control of NRF2 activity through increased NRF2 degradation. In this way, the regulation of NRF2 nuclear activity appears to be restricted to a particular circadian window, gated by both the clock-controlled mRNA synthesis and the KEAP1-based protein degradation.

Previous ChIP-seq (ChIP combined with deep sequencing) and microarray studies have revealed that, in addition to stress-induced antioxidant gene expression, NRF2 directly controls basal expression of numerous antioxidant genes via AREs in their promoters (Reddy et al. 2007; Malhotra et al. 2010). Here we show that, under physiological conditions in the lung, the protein levels and recruitment of NRF2 to gene-specific antioxidant gene promoters is under rhythmic clock regulation. *Nrf2*-deficient MEFs are not capable of maintaining circadian rhythms of a number of key antioxidant genes involved in glutathione homeostasis and show arrhythmic and reduced levels of its major antioxidant output glutathione. Moreover, in *Clock*^Δ19^ circadian mutant mice, the altered regulation of NRF2 activity was also associated with reduced expression of genes involved in glutathione metabolism as well as total glutathione levels. Since both the behavioral free-running rhythms in *Nrf2* knockout mice and *Dbp* mRNA rhythm in *Nrf2* knockout MEFs appear preserved, this highlights the essential role of NRF2 in mediating rhythmic glutathione homeostasis.

Changes in the lung NRF2 expression and GSH metabolism as well as elevated protein carbonyl content are recognized as central features of many human chronic lung diseases, including idiopathic pulmonary fibrosis, cystic fibrosis, acute respiratory distress syndrome, and COPD (Rahman and MacNee 2000; Dalle-Donne et al. 2003; Rangasamy et al. 2004; Reddy et al. 2007; Liu and Gaston Pravia 2009). *Nrf2* knockout mice show an increased propensity toward stress-induced tissue pathologies, including bleomycin-induced pulmonary fibrosis (Chan and Kan 1999; Cho et al. 2004). We observed temporal variations in protein carbonylation in wild-type mouse lungs, which were inversely correlated with changes in the reduced GSH levels. We show a time-of-day-dependent variation in the susceptibility to bleomycin-induced lung fibrosis in vivo, which was inversely correlated with the daily rhythm in the NRF2/GSH-dependent antioxidant response. Moreover, this time-dependent diurnal variation in sensitivity could be mitigated by the scheduled administration of the NRF2 activator SFN, highlighting the NRF2 pathway as a potential chronotherapeutic target to prevent or slow down the progression of oxidant-induced injury and fibrosis. In the lungs of *Clock*^Δ19^ circadian mutant mice, the altered temporal NRF2 activity was associated with constitutively lower levels of GSH, elevated protein oxidative damage, and a spontaneous fibrotic-like phenotype. We therefore speculate that decreased glutathione levels and GSH-dependent detoxification contribute to the increased oxidative/fibrotic damage in *Clock*^Δ19^ circadian mutant mice. Consistent with our findings, *Bmal1* knockout mice showed accelerated aging associated with constitutively high tissue ROS levels and oxidative stress-induced senescence (Kondratov et al. 2006, 2009), which could be rescued using glutathione precursor NAC (Khapre et al. 2011).

In conclusion, we identified the molecular circadian clock as an endogenous regulatory mechanism controlling the rhythmic activity of the redox-sensitive transcription factor NRF2 in the mouse lung. We also reveal the physiological significance of this rhythmic control in modulating tissue susceptibility to oxidative injury and pulmonary fibrosis. Given the role of NRF2 in human fibrotic diseases, we envisage a scenario in which circadian misalignments (e.g., caused by genetic variation or aging) may compromise the rhythmic activation of the NRF2-mediated antioxidant defense, leading to increased oxidative damage. Moreover, once fibrosis develops, there is no way to revert the phenotype. Current therapy that aims to slow down the progression of fibrotic diseases is often ineffective (Liu and Gaston Pravia 2009); our study implicates the need for considering circadian timing mechanisms, including timed drug administration (chronopharmacology) (Levi and Schibler 2007). Therefore, a major challenge in the future will be to understand how these circadian mechanisms affect tissue function in human disease.

## Materials and methods

### Animals and tissue collection

All experiments were conducted under the aegis of the 1986 Home Office Animal Procedures Act (UK). Mice were maintained on a standard maintenance chow under a 12-h light/12-h dark (12:12 LD) regimen. PER2∷Luc and Clock^Δ19^ mice on a C57BL/6J background were generated by Professor Joseph Takahashi. Clock^Δ19^ mice were subsequently bred with PER2−Luc mice. For circadian tissue collections or experiments with bleomycin-induced fibrosis, 8-wk-old female C57BL/6J mice (Harlan Laboratories) were placed under 12:12 LD cycles for 2 wk before their release into DD. Animals were sacrificed by cervical dislocation in complete darkness using an infrared viewer, and lung tissues were harvested at 4-h intervals, beginning at 39 h after the start of DD. All tissues (circadian and diurnal collections) were either freshly used or snap-frozen in liquid nitrogen and kept at −80°C until use.

### Reagents and plasmids

Bleomycin was purchased from ENZO. SFN was purchased from LKT Laboratories. All other chemicals were purchased from Sigma unless specified otherwise. mCLOCK and hBMAL1 expression plasmids and pGL3-m*Per1*∷luc were kind gifts from Dr. Kazuhiro Yagita. The lentiviral m*Per2*∷luc construct was previously described (Lu et al. 2010). The generation of wild-type and ΔE-box m*Nrf2*∷luc constructs is described in the Supplemental Material.

### Real-time bioluminescence recordings

Bioluminescence from the organotypic lung slices of wild-type and *Clock*Δ19 mice (on a PER2∷Luc background) (Yoo et al. 2004) or from MEFs with lentivirally transduced *Per2*∷luc reporters was recorded by photomultiplier tubes (PMTs; Hamamatsu) housed in a light-tight incubator at 37°C and 0% CO_2_ as described previously (Meng et al. 2008). The lung tissue slices were cultured in the recording medium (phenol red-free DMEM supplemented with 10% fetal bovine serum, 3.5 mg/mL glucose, 25 U/mL penicillin, 25 μg/mL streptomycin, 0.1 mM luciferin, 10 mM HEPES-NaOH at pH 7) on Millicell cell culture inserts (Millipore). Cellular clocks were synchronized by a well-established serum shock protocol using 50% horse serum (for 2 h). Data were presented as photon counts per minute.

### ChIP assays

Freshly harvested lung tissues were homogenized and cross-linked in 1% (v/v) formaldehyde. Lung nuclear lysates were sonicated (∼500 bp) using Bioruptor (Diagenode), and 5% of each sheared chromatin sample was saved as an input control. Following preclearing, lung chromatin was incubated overnight at 4°C with the following antibodies: 1 μg/mL rabbit anti-NRF2 (Santa Cruz Biotechnology), 4 μg/mL mouse anti-CLOCK (CLSP3) (Yoshitane et al. 2009; in-house), and 1 μg/mL control rabbit or mouse IgGs (Millipore). Immunoprecipitated DNA fragments were captured using protein G magnetic Dynabeads (Life Technologies). After a series of washes, proteinase K digestion, and reverse cross-linking, DNA was eluted and cleaned up (Qiagen). Real-time qPCR was performed using TaqMan-based primer pairs/probes (Supplemental Table 1) to amplify the promoter regions spanning the conserved E-box or AREs. The amount of precipitated DNA in each sample was quantified according to the percent input method, in which Ct values of each sample were normalized to the adjusted Ct values of their respective inputs (corrected for dilution factor) and expressed as percent input.

### Tissue glutathione (GSH) assay

Frozen lung tissues (100 mg) were homogenized in ice-cold 5% sulfosalicylic acid (Sigma) to minimize oxidation of reduced GSH. The diluted acid supernatants were used for GSH measurements, and the acid-precipitated protein was used for protein determination using a BCA protein assay kit (Pierce). Free reduced glutathione was determined using the luminescence-based GSH-Glow glutathione assay (Promega), which is based on the conversion of a luminogenic probe in the presence of reduced GSH in a reaction catalyzed by GST. Luminescence measurements were performed in triplicates for each biological replicate and processed in a single run to aid comparative quantification. Relative luminescence units (RLUs) were corrected for background luminescence and converted to nanomoles per milligram of protein using a standard curve for reduced GSH.

### Protein carbonylation assay

To measure protein carbonylation, lung protein lysates were processed using the Oxyblot protein oxidation detection kit (Millipore). Briefly, the carbonyl groups in the SDS-denatured proteins were derivatized to 2,3-dinitrophenyl hydrazone (DNP-hydrazone) by reaction with 2,4-dinitrophenylhydrazine (DNPH) followed by SDS-PAGE. Immunoblot detection of protein carbonyls was performed using rabbit anti-DNP primary antibody and goat anti-rabbit HRP-conjugated secondary antibody. As a negative control, proteins were derivatized using a derivatization control solution. Protein carbonylation levels were analyzed using National Institutes of Health ImageJ software by determining the values obtained for total protein density within each protein lane and were normalized to tubulin density for each sample.

### Bleomycin-induced lung fibrosis model

All animal procedures were carried out in accordance with the Animals Scientific Procedures Act (1986). Female wild-type (C57/BL6), PER2∷Luc, or clock^Δ19^ mice (aged 8–12 wk) were dosed intratracheally with 1 mg/kg clinical-grade bleomycin (30 µL volume; ENZO) or vehicle (saline) at either ZT0 (lights on) or ZT12 (lights off). Tissues were collected either 4 h later for RNA analysis of acute gene transcription or 7 d later (at ZT6) for histological assessment and long-term gene expression. The lungs were lavaged post-mortem via an incision in the trachea, with 1 mL of 10 mM EDTA and 1% BSA. For acute gene expression assays, fresh lung lobes were dissected and snap-frozen. For histological samples, 1 mL of 4% PFA was delivered via the trachea to inflate the lungs. The trachea was clamped off, and the lungs were removed en bloc and fixed in 4% PFA overnight at 4°C. After fixing, the middle right lobe was removed for RNA extraction, while the left lobe was processed for histological assessment. Lung slices at 5 μm were stained with Masson's Trichrome solution following the standard procedure within our core histology facilities (University of Manchester). The histological slides were imaged and scored numerically using the Ashcroft scoring system by two experienced researchers blinded to the treatment groups. For rescue studies, 10 mg/kg SFN (LKT Laboratories) or 10 mg/kg vehicle (saline) was administered intraperitoneally at ZT6 prior to a bleomycin challenge at ZT12 and then every other day for 7 d before tissue processing.

### Immunocytochemistry and immunohistochemistry

Immunostaining was performed as previously described (Pekovic et al. 2011). Briefly, cells were fixed with 4% formaldehyde at 12-h intervals after stimulation with 50% horse serum. For immunocytochemistry, rabbit anti-NRF2 antibody (sc-722, Santa Cruz Biotechnology) was applied at 1:200 overnight at 4°C. The secondary antibody was donkey anti-rabbit IgG conjugated to Alexa Fluor 555 (Molecular Probes). Nuclei were visualized by DAPI using VectaShield Hard-Set (Vector Laboratories). Cells on cover slips were imaged using a Zeiss Axiovert 40 CFL inverted microscope (Carl Zeiss). Intensity of NRF2 immunostaining was quantified using ImageJ software by measuring the integrated density of fluorescence in 50–100 cells after correcting for background fluorescence, and the results were confirmed in three independent experiments.

For immunohistochemistry, lung tissues were fixed with 4% formaldehyde, paraffin-embedded, and sectioned at 5 μm. Slides were dewaxed, rehydrated, and processed for antigen retrieval. Endogenous peroxidases were blocked in 3% H_2_O_2_ for 30 min. Blocking was carried out for 1 h in 2% normal goat serum, after which sections were incubated in anti-NRF2 antibody at 1:50 dilution overnight at 4°C in a humidified chamber. Slides were incubated in biotinylated secondary donkey anti-rabbit antibody (Vector Laboratories) diluted 1:800 in 1% BSA. ABC solution and the DAB (3′3′-diaminobenzidine) substrate kit (Vector Laboratories) were used to visualize positive NRF2 staining. To confirm the antibody specificity, immunohistochemistry was performed on control sections without the addition of either primary or secondary antibodies.

### Statistical analyses

Data were evaluated using Student's *t*-test, one-way ANOVA with Tukey test, or two-way ANOVA for multiple comparisons as indicated. Results are presented as mean ± SEM from at least three independent experiments. Differences were considered significant at the values of *P* < 0.05 (*), *P* < 0.01 (**), and *P* < 0.001 (***).

## References

[B1] Artaud-Macari E, Goven D, Brayer S, Hamimi A, Besnard V, Marchal-Somme J, Ali ZE, Crestani B, Kerdine-Römer S, Boutten A, 2013 Nuclear factor erythroid 2-related factor 2 nuclear translocation induces myofibroblastic dedifferentiation in idiopathic pulmonary fibrosis. Antioxid Redox Signal 18: 66–79 22703534 10.1089/ars.2011.4240

[B2] Ashcroft T, Simpson JM, Timbrell V 1988 Simple method of estimating severity of pulmonary fibrosis on a numerical scale. J Clin Pathol 41: 467–470 3366935 10.1136/jcp.41.4.467 PMC1141479

[B3] Beaver LM, Klichko VI, Chow ES, Kotwica-Rolinska J, Williamson M, Orr WC, Radyuk SN, Giebultowicz JM 2012 Circadian regulation of glutathione levels and biosynthesis in *Drosophila melanogaster*. PLoS ONE 7: e50454 23226288 10.1371/journal.pone.0050454 PMC3511579

[B4] Chan K, Kan YW 1999 Nrf2 is essential for protection against acute pulmonary injury in mice. Proc Natl Acad Sci 96: 12731–12736 10535991 10.1073/pnas.96.22.12731 PMC23072

[B5] Chen W, Sun Z, Wang XJ, Jiang T, Huang Z, Fang D, Zhang DD 2009 Direct interaction between Nrf2 and p21(Cip1/WAF1) upregulates the Nrf2-mediated antioxidant response. Mol Cell 34: 663–673 19560419 10.1016/j.molcel.2009.04.029 PMC2714804

[B6] Cho HY, Reddy SP, Yamamoto M, Kleeberger SR 2004 The transcription factor NRF2 protects against pulmonary fibrosis. FASEB J 18: 1258–1260 15208274 10.1096/fj.03-1127fje

[B7] Cho HY, Reddy SP, Kleeberger SR 2006 Nrf2 defends the lung from oxidative stress. Antioxid Redox Signal 8: 76–87 16487040 10.1089/ars.2006.8.76

[B8] Chowdhry S, Zhang Y, McMahon M, Sutherland C, Cuadrado A, Hayes JD 2013 Nrf2 is controlled by two distinct β-TrCP recognition motifs in its Neh6 domain, one of which can be modulated by GSK-3 activity. Oncogene 32: 3765–3781 22964642 10.1038/onc.2012.388 PMC3522573

[B9] Dalle-Donne I, Giustarini D, Colombo R, Rossi R, Milzani A 2003 Protein carbonylation in human diseases. Trends Mol Med 9: 169–176 12727143 10.1016/s1471-4914(03)00031-5

[B10] Edgar RS, Green EW, Zhao Y, van Ooijen G, Olmedo M, Qin X, Xu Y, Pan M, Valekunja UK, Feeney KA, 2012 Peroxiredoxins are conserved markers of circadian rhythms. Nature 485: 459–464 22622569 10.1038/nature11088 PMC3398137

[B11] Geyfman M, Kumar V, Liu Q, Ruiz R, Gordon W, Espitia F, Cam E, Millar SE, Smyth P, Ihler A, 2012 Brain and muscle Arnt-like protein-1 (BMAL1) controls circadian cell proliferation and susceptibility to UVB-induced DNA damage in the epidermis. Proc Natl Acad Sci 109: 11758–11763 22753467 10.1073/pnas.1209592109 PMC3406811

[B12] Gossan N, Zeef L, Hensman J, Hughes A, Bateman JF, Rowley L, Little CB, Piggins HD, Rattray M, Boot-Handford RP, 2013 The circadian clock in murine chondrocytes regulates genes controlling key aspects of cartilage homeostasis. Arthritis Rheum 65: 2334–2345 23896777 10.1002/art.38035 PMC3888512

[B13] Hastings MH, Reddy AB, Maywood ES 2003 A clockwork web: circadian timing in brain and periphery, in health and disease. Nat Rev Neurosci 4: 649–661 12894240 10.1038/nrn1177

[B14] Hayes JD, McMahon M 2009 NRF2 and KEAP1 mutations: permanent activation of an adaptive response in cancer. Trends Biochem Sci 34: 176–188 19321346 10.1016/j.tibs.2008.12.008

[B15] Higgins LG, Kelleher MO, Eggleston IM, Itoh K, Yamamoto M, Hayes JD 2009 Transcription factor Nrf2 mediates an adaptive response to sulforaphane that protects fibroblasts in vitro against the cytotoxic effects of electrophiles, peroxides and redox-cycling agents. Toxicol Appl Pharmacol 237: 267–280 19303893 10.1016/j.taap.2009.03.005

[B17] Itoh K, Wakabayashi N, Katoh Y, Ishii T, Igarashi K, Engel JD, Yamamoto M 1999 Keap1 represses nuclear activation of antioxidant responsive elements by Nrf2 through binding to the amino-terminal Neh2 domain. Genes Dev 13: 76–86 9887101 10.1101/gad.13.1.76 PMC316370

[B18] Itoh K, Mimura J, Yamamoto M 2010 Discovery of the negative regulator of Nrf2, Keap1: a historical overview. Antioxid Redox Signal 13: 1665–1678 20446768 10.1089/ars.2010.3222

[B20] Khapre RV, Kondratova AA, Susova O, Kondratov RV 2011 Circadian clock protein BMAL1 regulates cellular senescence in vivo. Cell Cycle 10: 4162–4169 22101268 10.4161/cc.10.23.18381 PMC3272294

[B21] Kikuchi N, Ishii Y, Morishima Y, Yageta Y, Haraguchi N, Itoh K, Yamamoto M, Hizawa N 2010 Nrf2 protects against pulmonary fibrosis by regulating the lung oxidant level and Th1/Th2 balance. Respir Res 11: 31 20298567 10.1186/1465-9921-11-31 PMC2846897

[B22] Ko CH, Takahashi JS 2006 Molecular components of the mammalian circadian clock. Hum Mol Genet 15: 271–277 10.1093/hmg/ddl207 16987893

[B23] Komatsu M, Kurokawa H, Waguri S, Taguchi K, Kobayashi A, Ichimura Y, Sou YS, Ueno I, Sakamoto A, Tong KI, 2010 The selective autophagy substrate p62 activates the stress responsive transcription factor Nrf2 through inactivation of Keap1. Nat Cell Biol 12: 213–223 20173742 10.1038/ncb2021

[B24] Kondratov RV, Kondratova AA, Gorbacheva VY, Vykhovanets OV, Antoch MP 2006 Early aging and age-related pathologies in mice deficient in BMAL1, the core component of the circadian clock. Genes Dev 20: 1868–1873 16847346 10.1101/gad.1432206 PMC1522083

[B25] Kondratov RV, Vykhovanets O, Kondratova AA, Antoch MP 2009 Antioxidant N-acetyl-L-cysteine ameliorates symptoms of premature aging associated with the deficiency of the circadian protein BMAL1. Aging 1: 979–987 20157581 10.18632/aging.100113 PMC2815755

[B26] Krishnan N, Davis AJ, Giebultowicz JM 2008 Circadian regulation of response to oxidative stress in *Drosophila melanogaster*. Biochem Biophys Res Commun 374: 299–303 18627767 10.1016/j.bbrc.2008.07.011 PMC2553425

[B27] Lai AG, Doherty CJ, Mueller-Roeber B, Kay SA, Schippers JH, Dijkwel PP 2012 CIRCADIAN CLOCK-ASSOCIATED 1 regulates ROS homeostasis and oxidative stress responses. Proc Natl Acad Sci 109: 17129–17134 23027948 10.1073/pnas.1209148109 PMC3479464

[B59] Lee J1, Moulik M, Fang Z, Saha P, Zou F, Xu Y, Nelson DL, Ma K, Moore DD, Yechoor VK 2013 Bmal1 and β-cell clock are required for adaptation to circadian disruption, and their loss of function leads to oxidative stress-induced β-cell failure in mice. Mol Cell Biol 33: 2327–2338 23547261 10.1128/MCB.01421-12 PMC3648066

[B28] Levi F, Schibler U 2007 Circadian rhythms: mechanisms and therapeutic implications. Annu Rev Pharmacol Toxicol 47: 593–628 17209800 10.1146/annurev.pharmtox.47.120505.105208

[B30] Liu RM, Gaston Pravia KA 2009 Oxidative stress and glutathione in TGF-β-mediated fibrogenesis. Free Radic Biol Med 48: 1–15 19800967 10.1016/j.freeradbiomed.2009.09.026 PMC2818240

[B31] Lu W, Meng QJ, Tyler NJ, Stokkan KA, Loudon AS 2010 A circadian clock is not required in an arctic mammal. Curr Biol 20: 533–537 20226667 10.1016/j.cub.2010.01.042

[B32] Malhotra D, Portales-Casamar E, Singh A, Srivastava S, Arenillas D, Happel C, Shyr C, Wakabayashi N, Kensler TW, Wasserman WW, 2010 Global mapping of binding sites for Nrf2 identifies novel targets in cell survival response through ChIP-seq profiling and network analysis. Nucleic Acids Res 38: 5718–5734 20460467 10.1093/nar/gkq212 PMC2943601

[B33] Malhotra D, Thimmulappa RK, Mercado N, Ito K, Kombairaju P, Kumar S, Ma J, Feller-Kopman D, Wise R, Barnes P, 2011 Denitrosylation of HDAC2 by targeting Nrf2 restores glucocorticosteroid sensitivity in macrophages from COPD patients. J Clin Invest 121: 4289–4302 22005302 10.1172/JCI45144 PMC3204828

[B34] McMahon M, Itoh K, Yamamoto M, Chanas SA, Henderson CJ, McLellan LI, Wolf CR, Cavin C, Hayes JD 2001 The cap'n'collar basic leucine zipper transcription factor Nrf2 (NF-E2 p45-related factor 2) controls both constitutive and inducible expression of intestinal detoxification and glutathione biosynthetic enzymes. Cancer Res 61: 3299–3307 11309284

[B35] McMahon M, Itoh K, Yamamoto M, Hayes JD 2003 Keap1-dependent proteasomal degradation of transcription factor Nrf2 contributes to the negative regulation of antioxidant response element-driven gene expression. J Biol Chem 278: 21592–21600 12682069 10.1074/jbc.M300931200

[B36] Meng QJ, McMaster A, Beesley S, Lu WQ, Gibbs J, Parks D, Collins J, Farrow S, Donn R, Ray D, 2008 Ligand modulation of REV-ERBα function resets the peripheral circadian clock in a phasic manner. J Cell Sci 121: 3629–3635 18946026 10.1242/jcs.035048 PMC3069549

[B37] Musiek ES, Lim MM, Yang G, Bauer AQ, Qi L, Lee Y, Roh JH, Ortiz-Gonzalez X, Dearborn JT, Culver JP, 2013 Circadian clock proteins regulate neuronal redox homeostasis and neurodegeneration. J Clin Invest 123: 5389–5400 24270424 10.1172/JCI70317 PMC3859381

[B38] Nguyen T, Nioi P, Pickett CB 2009 The Nrf2-antioxidant response element signaling pathway and its activation by oxidative stress. J Biol Chem 284: 13291–13295 19182219 10.1074/jbc.R900010200 PMC2679427

[B60] O'Neill JS, van Ooijen G, Dixon LE, Troein C, Corellou F, Bouget FY, Reddy AB, Millar AJ 2011 Circadian rhythms persist without transcription in a eukaryote. Nature 469: 554–558 21270895 10.1038/nature09654 PMC3040569

[B39] Panda S, Antoch MP, Miller BH, Su AI, Schook AB, Straume M, Schultz PG, Kay SA, Takahashi JS, Hogenesch JB 2002 Coordinated transcription of key pathways in the mouse by the circadian clock. Cell 109: 307–320 12015981 10.1016/s0092-8674(02)00722-5

[B40] Patel SA, Velingkaar N, Kondratov RV 2014 Transcriptional control of antioxidant defense by the circadian clock. Antioxid Redox Signal doi: 10.1089/ars.2013.5671 10.1089/ars.2013.5671 PMC4038985 24111970

[B41] Peek CB, Affinati AH, Ramsey KM, Kuo HY, Yu W, Sena LA, Ilkayeva O, Marcheva B, Kobayashi Y, Omura C, 2013 Circadian clock NAD^+^ cycle drives mitochondrial oxidative metabolism in mice. Science 342: 1243417 24051248 10.1126/science.1243417 PMC3963134

[B42] Pekovic V, Gibbs-Seymour I, Markiewicz E, Alzoghaibi F, Benham AM, Edwards R, Wenhert M, von Zglinicki T, Hutchison CJ 2011 Conserved cysteine residues in the mammalian lamin A tail are essential for cellular responses to ROS generation. Aging Cell 10: 1067–1079 21951640 10.1111/j.1474-9726.2011.00750.x

[B43] Rahman I, MacNee W 2000 Oxidative stress and regulation of glutathione in lung inflammation. Eur Respir J 16: 534–554 11028671 10.1034/j.1399-3003.2000.016003534.x

[B44] Rangasamy T, Cho CY, Thimmulappa RK, Zhen L, Srisuma SS, Kensler TW, Yamamoto M, Petrache I, Tuder RM, Biswal S 2004 Genetic ablation of Nrf2 enhances susceptibility to cigarette smoke-induced emphysema in mice. J Clin Invest 114: 1248–1259 15520857 10.1172/JCI21146 PMC524225

[B45] Reddy NM, Kleeberger SR, Yamamoto M, Kensler TW, Scollick C, Biswal S, Reddy SP 2007 Genetic dissection of the Nrf2-dependent redox signaling-regulated transcriptional programs of cell proliferation and cytoprotection. Physiol Genomics 32: 74–81 17895394 10.1152/physiolgenomics.00126.2007

[B46] Reppert SM, Weaver DR 2002 Coordination of circadian timing in mammals. Nature 418: 935–941 12198538 10.1038/nature00965

[B47] Rey G, Cesbron F, Rougemont J, Reinke H, Brunner M, Naef F 2011 Genome-wide and phase-specific DNA-binding rhythms of BMAL1 control circadian output functions in mouse liver. PLoS Biol 9: e1000595 21364973 10.1371/journal.pbio.1000595 PMC3043000

[B48] Ripperger JA, Schibler U 2006 Rhythmic CLOCK–BMAL1 binding to multiple E-box motifs drives circadian Dbp transcription and chromatin transitions. Nat Genet 38: 369–374 16474407 10.1038/ng1738

[B49] Stangherlin A, Reddy AB 2013 Regulation of circadian clocks by redox homeostasis. J Biol Chem 288: 26505–26511 23861436 10.1074/jbc.R113.457564 PMC3772198

[B51] van der Horst GT, Muijtjens M, Kobayashi K, Takano R, Kanno S, Takao M, de Wit J, Verkerk A, Eker AP, van Leenen D, 1999 Mammalian Cry1 and Cry2 are essential for maintenance of circadian rhythms. Nature 398: 627–630 10217146 10.1038/19323

[B52] Veal EA, Day AM, Morgan BA 2007 Hydrogen peroxide sensing and signaling. Mol Cell 26: 1–14 17434122 10.1016/j.molcel.2007.03.016

[B53] Vitaterna MH, King DP, Chang AM, Kornhauser JM, Lowrey PL, McDonald JD, Dove WF, Pinto LH, Turek FW, Takahashi JS 1994 Mutagenesis and mapping of a mouse gene, Clock, essential for circadian behavior. Science 264: 719–725 8171325 10.1126/science.8171325 PMC3839659

[B54] Walters DM, Kleeberger SR 2008 Mouse models of bleomycin-induced pulmonary fibrosis. Curr Protoc Pharmacol 40: 5.46.1–5.46.17 10.1002/0471141755.ph0546s40 22294226

[B55] Wang TA, Yu YV, Govindaiah G, Ye X, Artinian L, Coleman TP, Sweedler JV, Cox CL, Gillette MU 2012 Circadian rhythm of redox state regulates excitability in suprachiasmatic nucleus neurons. Science 337: 839–842 22859819 10.1126/science.1222826 PMC3490628

[B56] Xu YQ, Zhang D, Jin T, Cai DJ, Wu Q, Lu Y, Liu J, Klaassen CD 2012 Diurnal variation of hepatic antioxidant gene expression in mice. PLoS ONE 7: e44237 22952936 10.1371/journal.pone.0044237 PMC3430632

[B57] Yoo SH, Yamazaki S, Lowrey PL, Shimomura K, Ko CH, Buhr ED, Siepka SM, Hong HK, Oh WJ, Yoo OJ, 2004 PERIOD2:LUCIFERASE real-time reporting of circadian dynamics reveals persistent circadian oscillations in mouse peripheral tissues. Proc Natl Acad Sci 101: 5539–5546 10.1073/pnas.0308709101 PMC397382 14963227

[B58] Yoshitane H, Takao T, Satomi Y, Du NH, Okano T, Fukada Y 2009 Roles of CLOCK phosphorylation in suppression of E-box-dependent transcription. Mol Cell Biol 29: 3675–3686 19414601 10.1128/MCB.01864-08 PMC2698759

